# Eccrine Hidradenocarcinoma of the Scalp

**DOI:** 10.7759/cureus.23023

**Published:** 2022-03-10

**Authors:** Diana Martins, Filipa Pereira, Rosa Azevedo, Ivo Julião

**Affiliations:** 1 Medical Oncology, Instituto Português de Oncologia do Porto Francisco Gentil, EPE, Porto, PRT; 2 Medical Oncology, Hospital de Braga, EPE, Braga, PRT; 3 Pathology, Instituto Português de Oncologia do Porto Francisco Gentil, EPE, Porto, PRT

**Keywords:** hidradenocarcinoma, eccrine, oncology, skin cancer, sweat gland neoplasm

## Abstract

Eccrine hidradenocarcinoma is a rare adnexal neoplasm arising from the eccrine sweat glands of the skin. Surgery and radiotherapy are the mainstay of treatment, and chemotherapy is reserved for unresectable or metastatic lesions. We present the case of a 60-year-old man, referred for treatment of an unresectable basal cell carcinoma of the scalp. He started Vismodegib in January of 2017 but progressed after three months. A new biopsy showed a poorly differentiated carcinoma. The patient started carboplatin/paclitaxel with a major response, enabling surgery in December of 2017. Pathology concluded on a hidradenocarcinoma, R1, and radiotherapy was not possible due to local infection. Four months later, he underwent radiotherapy due to local recurrence, and restarted carboplatin/paclitaxel, but with progressive disease. An exploratory surgery in October of 2018 revealed unresectable disease. Restaging showed lung metastasis and second-line chemotherapy was proposed. However, due to continued clinical deterioration, the patient died in February of 2019.

## Introduction

Hidradenocarcinoma (HC) is a rare malignant adnexal tumour that accounts for approximately 6% of malignant eccrine tumours and less than 0.001% of all tumours [1-3]. Malignant adnexal tumours arise from the pilosebaceous unit, eccrine and apocrine glands and are a heterogeneous group of uncommon tumours, with different biological behaviours [2,4]. Tumour classification is complex, which is worsened by the existence of controversial literature on the subject. HC is usually a tumour of eccrine origin that often arises de novo but may also result from malignant transformation of a pre-existing hidradenoma [2,5]. It was described for the first time by Keasby et al. in 1954 [6]. Its incidence peaks in the fifth to seventh decade, with a mean age of 50 years, and arises mostly in the head and neck region [1-4,7-9]. However, it can potentially develop anywhere in the skin [2]. Some series have shown equal incidences between sex, while others described a slight female predominance [2,4,10,11]. Macroscopic evaluation of the lesion is not enough for diagnosis and therefore, histology and immunohistochemistry (IHC) are essential [9]. HC is an aggressive tumour, with recurrence rates after surgery around 50% [2]. Up to 60% of patients will present distant metastases within the first two years, with low survival rates (5-year postsurgical survival rate is reported to be <30%) [3]. Multiple nodules, large lesions, and lymph node involvement are poor prognostic factors [1-4,7-9].

There are no randomized trials on HC treatment and tumour board (TB) decisions are usually made based on retrospective series, case reports, or institutional experience. However, it is generally accepted that wide resection surgery is the optimal approach and, due to the high rates of local recurrence, adjuvant radiotherapy (RT) can be considered [11]. Systemic chemotherapy (ChT) is reserved for unresectable or metastatic disease, but responses are usually poor [1,3,7,9].

## Case presentation

**Figure 1:**
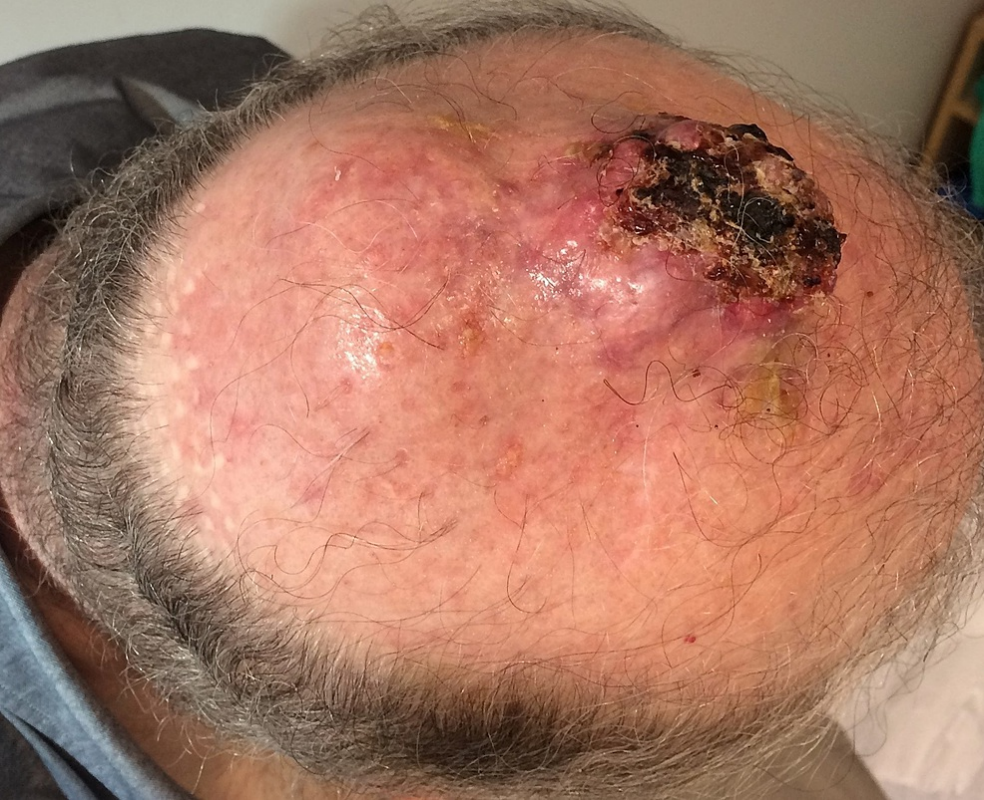
Scalp lesion on admission in our Institution

We report the case of a 60-year-old man with a past medical history of hypertension and dyslipidemia, who in January of 2015 was submitted to the excision of a nodule of the scalp. The pathology report concluded on a basal cell carcinoma. One year later, the lesion recurred and was, again, removed. Pathology was consistent: infiltrative and ulcerative basal cell carcinoma with a thickness of 10 mm and a positive deep margin. Two months later, the lesion regrown and increased rapidly. A new biopsy confirmed the initial diagnosis and the patient was then referred to our Institution. The patient presented two verrucous and ulcerated nodules, with haemorrhagic crust, in the parieto-occipital region of the scalp, with 5 cm of largest diameter (Figure 1).

**Figure 2:**
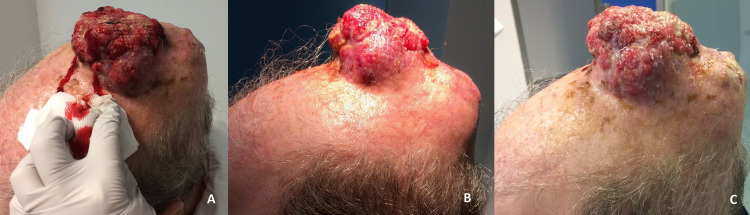
Scalp lesion after three months of Vismodegib treatment

The TB proposed systemic treatment after considering the lesion unresectable and inadequate for RT. The patient started Vismodegib in January of 2017 and, three months later, the lesion was largely progressing (Figure 2A-2C). Due to the unexpected response and behaviour, the lesion was again submitted to biopsy, this time at our institution. Surprisingly, pathology concluded on a poorly differentiated carcinoma and staging presented no metastatic lesions.

**Figure 3:**
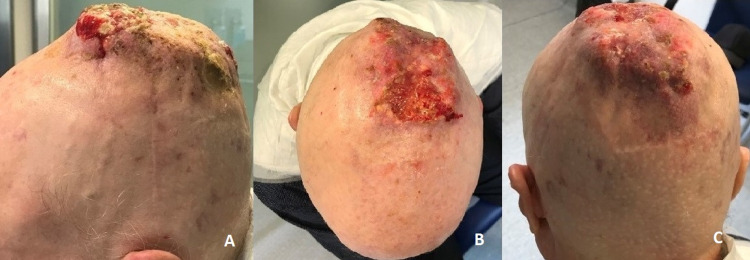
A, B, C - Partial response to chemotherapy

Upon the shift on diagnosis, the patient was now proposed for ChT and started carboplatin (AUC5) and paclitaxel (175mg/m2) q3w in May of 2017. He completed 6 cycles with very good partial response (Figure 3) and large volume reduction.

**Figure 4:**
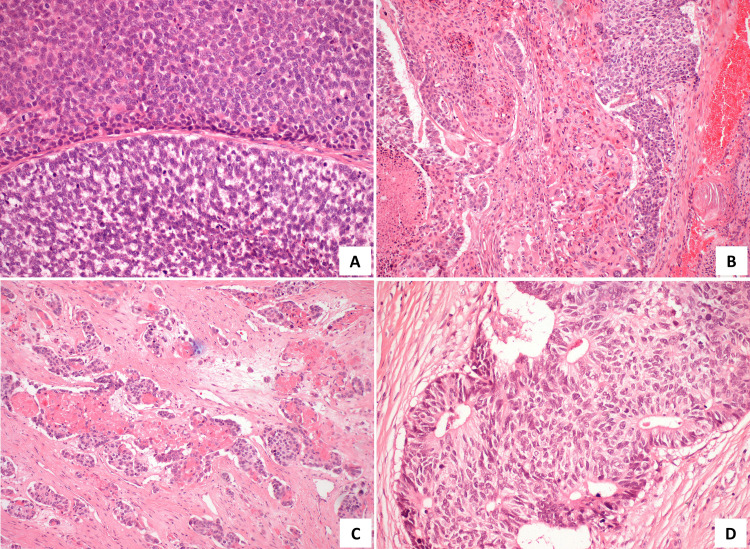
Photomicrographs of the tumor. A - Clear and basaloid cells (H&E, 200x); B - Foci of epidermoid differentiation (H&E, 100x); C - Malignant cells arranged in solid sheets, variably sized nodules with sclerotic stroma (H&E, 100x); D - Glandular differentiation (H&E, 200x).

**Figure 5:**
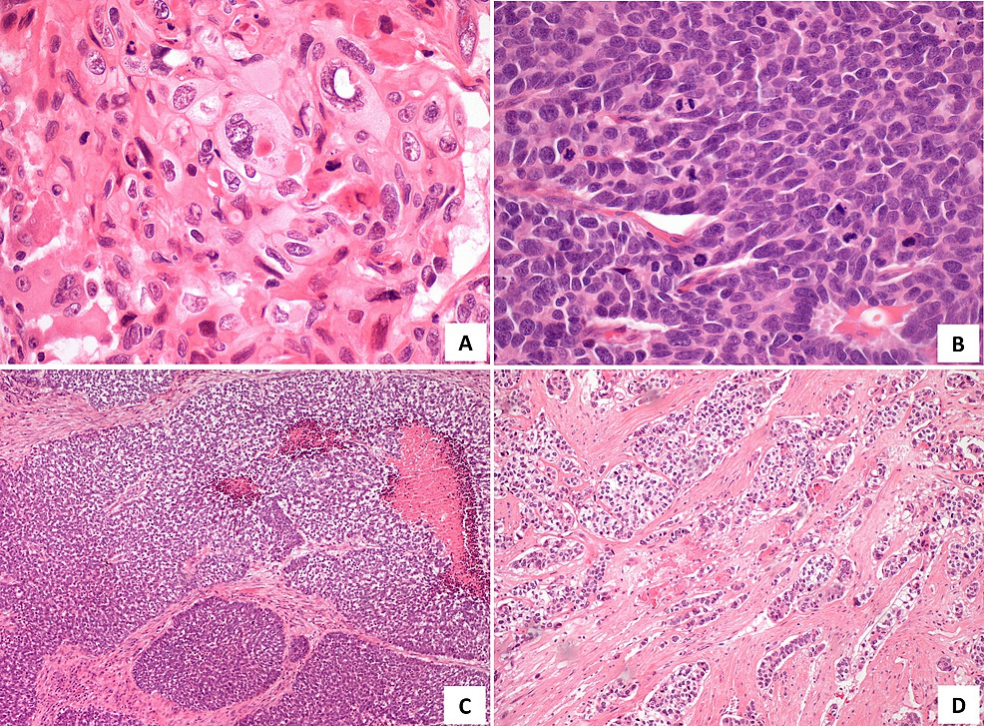
Photomicrographs of the tumor. A - Nuclear pleomorphism (H&E, 400x); B - Increased mitotic activity (H&E, 400x); C - Necrosis (H&E, 100x); D - Infiltrative growth pattern (H&E stain, 100x)

**Figure 6:**
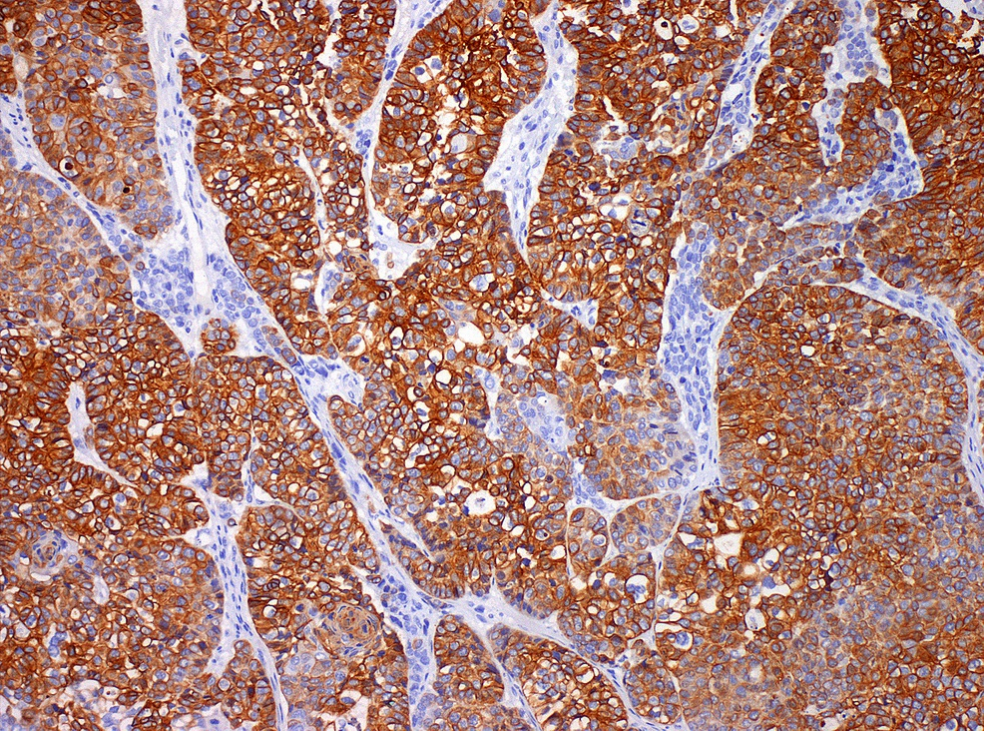
Immunohistochemical staining positive for CK7, 100x

**Figure 7:**
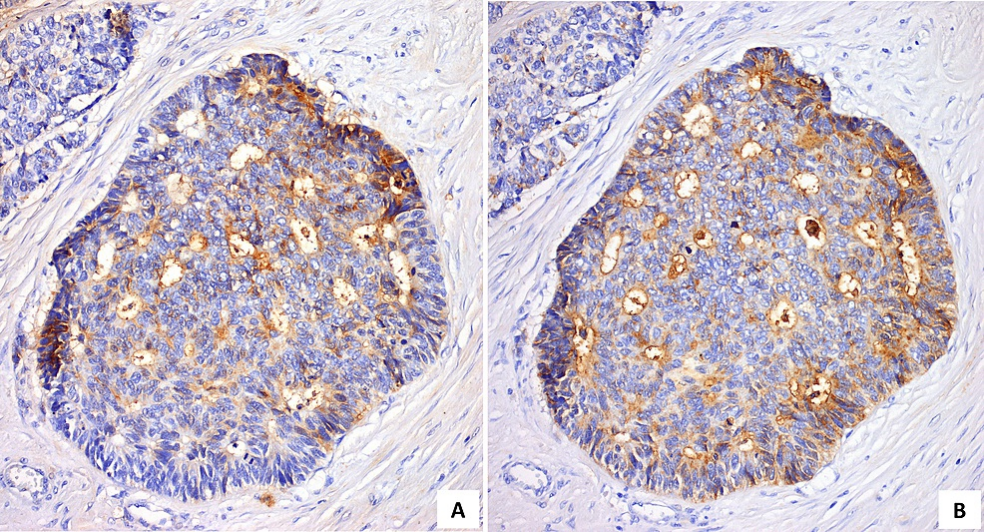
Immunohistochemical staining positive for CEA (A; 200x) and EMA (B; 200x)

The good response led the TB to propose surgery. Thus, in November of 2017, he was submitted to an extended excision of the lesion and bone, reconstructed with a dorsal muscle flap. In December of 2017, he underwent remodeling and cranioplasty with a methyl methacrylate prosthesis. The final histology concluded on a malignant hidradenocarcinoma showing variable cellular composition and cytologic grade. Cellular heterogeneity consisted of clear cells (Figure 4A), squamoid cells (Figure 4B), mucinous cells, oncocytic cells, basaloid cells, and transitional elements. The neoplastic cells were arranged in solid sheets, variably sized nodules with sclerotic stroma (Figure 4C), and glandular differentiation (Figure 4D). The malignant cells showed nuclear pleomorphism (Figure 5A), increased mitotic activity (Figure 5B), necrosis (Figure 5C), and infiltrative growth pattern (Figure 5D). A positive resection margin was identified at the deep border. On immunohistochemical staining, the cells expressed CK7 (Figure 6) and stained positively for CEA (Figure 7A) and EMA (Figure 7B). The results for p53 were negative and Ki67 was 90%. The patient was proposed for adjuvant RT, which he did not start due to surgical wound infection and, therefore, started surveillance.

**Figure 8:**
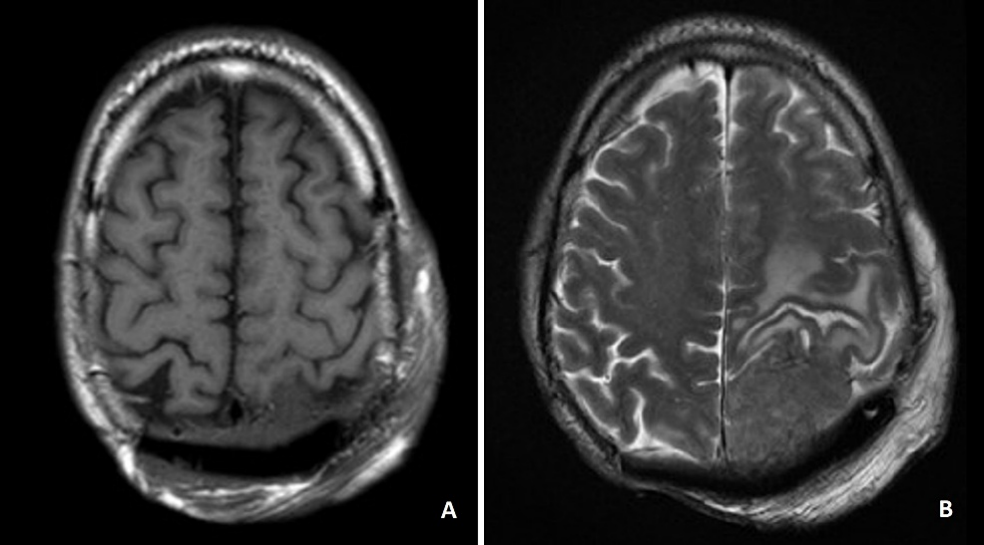
A - Brain MRI (axial, T2); parietal lesion compressing the brain parenchyma; B - Brain MRI (axial, T2); disease progression on chemotherapy

In April of 2018, a follow-up brain MRI (Figure 8A) showed an extra-axial parietal lesion with 15x15x19 mm, compressing the brain and the TB proposed palliative RT. However, before starting treatment, he presented with neurological complaints (mainly motor discoordination) and was admitted to the emergency department. A brain CT showed an extra-axial left parietal lesion with 38 x 27 mm. He then underwent whole-brain radiotherapy in May of 2018, with clinical recovery and improvement of performance status. He restarted the same ChT regimen. After three cycles, he had a syncope and brain MRI showed disease progression (expansive left parietal lesion with 6 cm with mass effect on brain parenchyma and left lateral ventricle) (Figure 8B). The TB proposed exploratory surgery and, in October of 2018, the patient was submitted to parietal-occipital craniotomy with extended excision of extensive infiltrative lesion that invaded the parenchyma, dura-mater, and superior sagittal sinus and was finally deemed unresectable. This was followed by plasty with a pectoral skin flap. At this time, a staging CT showed multiple bilateral lung metastases and he was proposed for palliative treatment with capecitabine. However, the patient presented with clinical deterioration and did not start treatment, dying in February of 2019.

## Discussion

Hidradenocarcinoma is a rare and aggressive malignancy. Clinically, it usually presents as a solitary, firm, intradermal nodule, covered by intact or ulcerated skin. Most of the patients remain asymptomatic, but some of them can present discomfort, pain, ulceration, or bleeding [1,2,7,9]. It can slowly grow over several years, but it may experience a sudden increase in size and this may possibly be related to a malignant transformation from hidradenoma [1,9]. Most frequently, it appears in the skin of the head and neck, especially on the face, and more rarely on the distal extremities, trunk, abdomen, groin, and scalp [2,9]. Differential diagnosis using macroscopy is very difficult due to the morphological similarities with other entities, namely benign lesions. Microscopically, HC is composed of the same cell types as hidradenoma but differs by presenting high nuclear pleomorphism, necrosis, increased mitotic rate, infiltrative growth pattern, and perineural and lymphovascular invasion [4]. There are no specific immunohistochemical features that establish the diagnosis, but some markers support it. Tumour cells often stain positively for CK5/6 and luminal surfaces of ductal structures may be highlighted by CEA and EMA [2,3].

There are no randomized trials or consensus on HC treatment which is currently based on retrospective series, clinical cases, or institutional experience. However, surgery with wide margins is considered the mainstay of treatment. Some series suggest at least 2 cm margins, but more recently 3-5 cm are advised [7,9]. However, survival rates at five years are less than 30% [1-4,7-9]. Despite being controversial, RT may be considered as an adjuvant treatment to improve local control, especially in patients with high-risk features: deep structure invasion, positive margins, highly anaplastic morphology, and extracapsular lymph node extension, with retrospective overall survivals of 27-35 months [10,11]. If the tumour is unresectable, RT using high doses (50-70 Gy) is also possible [2-3,7,10-11]. In the absence of known distant metastases, clinically involved lymph nodes should be dissected [2,8-10].

Currently, ChT has no role in the adjuvant setting, neither alone nor in combination with RT, but it can be an approach for metastatic or unresectable disease. Many ChT regimens have been used with varying response rates. First-line ChT usually uses fluoropyrimidines-based regimens such as 5-fluorouracil or capecitabine, but others are shown to be effective such as carboplatin and paclitaxel. Second-line agents can include the previous or doxorubicin, platins, cyclophosphamide, vincristine, and bleomycin, but with poor objective responses and short survivals [1-3, 7, 9].

Thus, HC is a rare and aggressive tumour, with limited treatment options and poor survival. Sharing this clinical case may help in the early recognition of this disease, which is critical for a better prognosis. The inclusion of these patients in clinical trials is warranted, albeit difficult, due to its low incidence. However, in the era of molecular approaches to treatment, basket or umbrella trials could be a sound option to develop evidence for the treatment of this dismal disease.

## Conclusions

Hidradenocarcinoma is a rare and aggressive malignancy. Differential diagnosis is challenging due to the morphological similarities with other entities. There is no consensus regarding treatment, but surgery is considered the mainstay and RT may be considered to improve local control. ChT could be an approach for metastatic or unresectable disease, but with poor response rates. Further research is needed to elucidate the most appropriate treatment strategies and improve the overall prognosis. 
